# Association between sporting event attendance and self-rated health: an analysis of multiyear cross-sectional national data in Japan

**DOI:** 10.1186/s41256-018-0068-9

**Published:** 2018-05-06

**Authors:** Yuhei Inoue, Mikihiro Sato, Makoto Nakazawa

**Affiliations:** 1 School of Kinesiology, University of Minnesota, 218 Cooke Hall, 1900 University Ave. SE, Minneapolis, MN 55455 USA; 2 Hart School of Hospitality, Sport and Recreation Management, James Madison University, Godwin Hall 355, MSC 2305, Harrisonburg, VA 22807 USA; 3 Faculty of Health and Sport Sciences, University of Tsukuba, 1-1-1 Tennodai, Tsukuba, Ibaraki, 305-8574 Japan

**Keywords:** Leisure, Population health, Secondary data, Spectatorship, Spectator sport, Sport spectating, Well-being

## Abstract

**Background:**

This study examined the extent to which sporting event attendance is associated with self-rated health. Drawing from an economic model of health production and psychological research on the health benefits of psychosocial resources, sporting event attendance was hypothesized to have a positive relationship with self-rated health.

**Methods:**

A two-level multilevel ordered logistic regression was used to analyze multiyear cross-sectional data collected from national surveys in Japan.

**Results:**

The results demonstrate that, controlling for the effects of personal and environmental characteristics, sporting event attendance positively correlates with self-rated health over a 12-year period. Specifically, when compared to individuals who did not attend any sporting event during the past year, those who attended a sporting event were 33% more likely to indicate a higher level of self-rated health.

**Conclusions:**

These findings provide evidence for a positive association between sport spectatorship and the perception of general health and contribute to the literature examining the relationship between sport spectatorship and health outcomes.

## Background

Attending live sporting events is a popular form of leisure. In 2014, the total attendance for major sporting events across the world exceeded 400 million, with professional and college sporting events in North America alone attracting over 220 million spectators [1]. In Australia, over 40% of the population attended at least one sporting event in 2010 [2]. Although the contribution of leisure to health has been identified primarily for physically active leisure [3–5], evidence supports the notion that participating in *nonexercise* forms of leisure, such as attending cultural events and engaging in arts, also correlates to better health [6, 7]. Leisure as a concept entails much more than just physical exercise [6]. Nonexercise leisure can engage a wide range of the population, including those who are not physically active. Therefore, understanding the health benefits of sporting event attendance, which represents an important form of nonexercise leisure, has broad implications for public health policy and practice. As such, the purpose of this research is to determine the extent to which sporting event attendance correlates with self-rated health—a subjective assessment of global health status [8].

Grossman’s [9] health production model provides the theoretical basis for this research. This model assumes that people are born with an initial stock of health. As they age, this stock depreciates at an increasing rate, with death occurring “when the stock falls below a certain level” [9]. This depreciation process, however, does not happen equally for all people. Rather, individuals are capable of increasing their length of life through their efforts to attain health resources, such as medical care and healthy lifestyle choices [9, 10]. Based on Grossman’s health production model, previous studies examined the effects of leisure activities, especially physical activity, on health, finding that leisure participation is associated with enhanced health status as measured by self-rated health [4, 10].

Based on Grossman’s [9] health production model, our key assertion is that attending a live sporting event as spectators represents a lifestyle behavior that allows individuals to increase health resources and hence enjoy good health as indicated by an elevated level of self-rated health. This assertion finds support from the psychology literature that highlights the role of psychosocial resources in maintaining and enhancing health outcomes [11, 12]. Specifically, the literature posits that the extent to which individuals can maintain good health is at least partly determined by the availability of psychological resources (e.g., positive emotions, self-esteem) and social resources (e.g., social support, social capital). This is because these resources can serve as protective factors that reduce the negative health effects of stressful events and adversity [11, 12]. Consistent with this psychological approach to understanding health, researchers have demonstrated that leisure represents a context in which individuals obtain various psychosocial resources, such as a sense of meaningfulness, positive emotions, resilience, and friendships and companionships [13].

As a leisure activity, sporting event attendance has been shown to provide people with various forms of psychological and social resources [14–18]. For example, a qualitative study of fans of an English football team found that attending the team’s games served as a temporary escape from daily life and helped fans reduce the negative effects of stress [14]. In a survey with supporters of English football teams, respondents described their team’s home stadium as a place that feels like home and that allows them to interact positively with other supporters [19]. Moreover, a study of spectators of Japanese professional football games found that the spectators’ psychological connection with hometown teams was positively associated with their perceptions of social support as well as social cohesion in their communities [15].

The collective evidence from the abovementioned studies suggests that attending live sporting events may produce psychosocial resources. The creation of these resources through sporting event attendance, in turn, could help reduce the negative health consequences of stress and adversity [11, 12] and serve as a general protective factor [13], which would slow down the depreciation of the stock of health [9]. It is important to acknowledge empirical evidence indicating that sport spectating could have adverse effects on health in the short term, such as temporal experience of psychological distress and engagement in unhealthy eating habits [17, 20]. According to Grossman’s [9] health production model, however, such short-term negative effects can be outweighed by the long-term health benefits of sporting event attendance. That is, even though the psychological distress caused by watching their team’s loss at a sporting event may negatively influence the health of spectators in the short term, the psychosocial resources they develop from the event, such as friendships with other spectators, endure over time and benefit their health in the long run [4, 9, 10]. Consequently, we hypothesized that sporting event attendance has a positive relationship with self-rated health.

## Methods

### Data

Our hypothesis was tested using secondary data from the National Sports-Life Survey (NSLS), a national survey conducted every two years in Japan since 1992 to examine trends in sport participation and health status among adults. In the NSLS, a representative sample of the adult Japanese population aged 20 and older was selected by a two-stage sampling method. In the first sampling stage, all municipalities in Japan were classified into 11 prefecture-based regions. The regions were then classified into 44 areas according to four urban-size categories (metropolitan cities, cities with a population of 100,000 or more, cities with a population below 100,000, and rural towns and villages). In the second stage, the 44 areas were further divided into 210 observation spots in proportion to the population size of each area. Approximately 9 to 16 adults per observation spot were selected to participate in the survey.

The NSLS included different sets of survey questions for each year the survey was conducted. During the period of 2000 to 2012, the NSLS was conducted seven times in total (in 2000, 2002, 2004, 2006, 2008, 2010, and 2012), but the survey did not include a measure of self-rated health in 2002, 2008, and 2010. We therefore tested the hypothesis by focusing on data from the four years (2000, 2004, 2006, and 2012) that included consistent measures for self-rated health.^1^

**Fig. 1:**
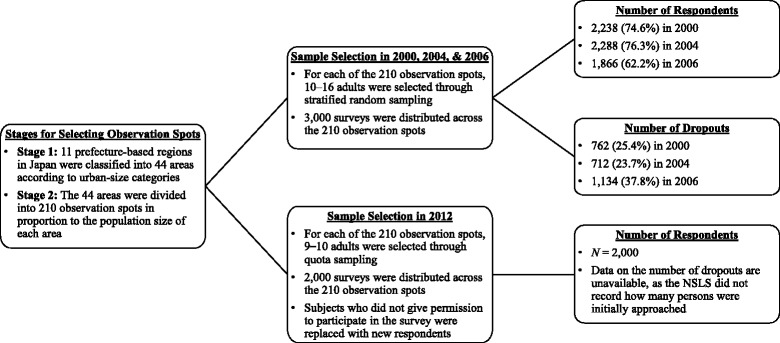
Procedures for Selecting Study Participants

Figure 1 describes the procedures used for selecting study participants for each of these four years. In 2000, 2004, and 2006, surveys were distributed to a total of 3000 adults selected from the 210 observation spots through stratified random sampling. In 2000, 2238 (74.6%) returned usable responses; in 2004, 2288 (76.3%) returned usable responses; and in 2006, 1866 (62.2%) returned usable responses.

In 2012, 2000 surveys were distributed through quota sampling, in which the proportion of respondents from each observation spot was matched to the proportion of the adult Japanese population in terms of age groups and gender. When initial subjects did not give permission to participate in the survey, data collectors (trained and hired by a private market research company) replaced these individuals with new respondents who resided in the same observation spot and had the same demographic characteristics (gender, age group) to reach the predetermined number of usable responses for each spot. Through this procedure, 2000 usable responses were collected in 2012.

The original NSLS datasets included all usable responses collected for each year. Of these responses, we removed responses (17 in 2000, 15 in 2004, 13 in 2006, and 6 in 2012) that contained missing values for one or more of our study variables (see the next section) before analyzing the data to test the hypothesis. Given that the exclusion of responses due to missing values was small across the four years (less than 1%), listwise deletion was considered appropriate for the current study [21]. The final sample size for each of the four years used for the current study is as follows: 2221 for Year 2000, 2273 for Year 2004, 1853 for Year 2006, and 1994 for Year 2012.

### Measures

#### Self-rated health

Self-rated health, our dependent variable, was assessed by a single item asking respondents to rate their current state of general health on a 4-point scale including *poor* (1), *fair* (2), *good* (3), and *very good* (4). A 4-point scale measure of self-rated health has been frequently used in past studies and was shown to predict objective health indicators, such as mortality [22, 23].

#### Sporting event attendance

In the NSLS, respondents were asked to indicate whether they had attended at least one sporting event during the past year. Based on this information, we created a dummy variable of sporting event attendance (1 = those who attended one or more sporting events during the past year; 0 = those who did not attend any sporting event), which served as the independent variable for this study. Measuring sporting event attendance as a dummy variable is consistent with previous research that used dichotomous variables of various leisure activities to examine the relationship between leisure participation and health outcomes [24, 25].

#### Control variables

To take into account the potential effects of personal and environmental characteristics on self-rated health [22], the analysis included several control variables available in NSLS for all four years. For personal characteristics, the following dummy variables were included: male (1 = male respondents), living with a spouse (1 = respondents who lived with a spouse during the time of data collection), and unemployment (1 = respondents who were unemployed during the time of data collection). The analysis also included two continuous variables: age (respondents’ self-reported age) and sport participation level (respondents’ level of sport participation during the past year assessed on a 5-point scale from 1 [*did not participate in any sport activity*] to 5 [*participated in at least 30 min of vigorous sport activity more than twice a week*]). For environmental characteristics, we included a variable representing the size of the city where respondents lived, measured on a 4-point scale (1 = rural towns and villages; 2 = cities with a population below 100,000; 3 = cities with a population of 100,000 or more; 4 = metropolitan cities).

### Analysis

A two-level multilevel ordered logistic regression analysis was used to examine the association between sporting event attendance and self-rated health [26]. As noted above, in the original NSLS sampling process, all municipalities in Japan were classified into 11 prefecture-based regions. However, when the regional variable was created in the NSLS data, these regions were condensed, according to proximity and shared regional characteristics, into the eight regions (Hokkaido, Tohoku, Kanto, Chubu, Kinki, Chugoku, Shikoku, Kyushu) that reflect the most common regional classification in Japan based on history, cultural characteristics, and administrative needs [27]. Using this regional classification, we performed five multilevel regression models with individuals nested within the eight regions to examine the association for each of the 4 years and for the 4 years combined. For each model, self-rated health was regressed on sporting event attendance and all control variables described above. All multilevel regression analyses were conducted using Stata, version 14.

## Results

Table 1 presents the descriptive statistics for each of the 4 years and for the 4 years combined. Over 70% of the respondents rated their health as good or very good for all 4 years. The percentage of the respondents attending at least one sporting event during the past year fell within the narrow range of 30% (2006) to 37% (2004), with a 4-year combined average of 33%. The sample from each year also provided similar values for the other personal and geographical variables examined. The consistency of sample characteristics across the 4 years supported the legitimacy of comparing the association between sporting event attendance and self-rated health for these years.

**Table 1:** Descriptive Statistics

Variable	Mean (SD) for continuous variables / Percentage of each category for self-rated health and of yes for binary variables				
	2000	2004	2006	2012	4 years combined
	(*N* = 2221)	(*N* = 2273)	(*N* = 1853)	(*N* = 1994)	(*N* = 8341)
Self-rated health					
Poor (1)	5.13%	4.80%	6.04%	3.76%	4.92%
Fair (2)	11.84%	24.95%	20.83%	20.31%	19.43%
Good (3)	63.39%	62.69%	64.98%	67.35%	64.50%
Very good (4)	19.63%	7.57%	8.15%	8.58%	11.15%
Sporting event attendance	32.69%	37.22%	29.95%	31.70%	33.08%
Age	48.17 (15.72)	49.59 (16.70)	50.89 (17.14)	49.47 (16.71)	49.47 (16.57)
Male	49.21%	49.36%	48.19%	49.50%	49.09%
Living with a spouse	75.10%	72.00%	69.35%	72.77%	72.41%
Unemployment	12.16%	16.28%	17.22%	14.54%	14.97%
Sport participation level	1.62 (1.48)	1.71 (1.45)	1.60 (1.48)	1.84 (1.50)	1.69 (1.48)
City size	2.55 (1.06)	2.63 (1.05)	2.66 (1.02)	2.88 (0.93)	2.67 (1.02)
Region: Hokkaido	4.72%	4.97%	4.32%	4.76%	4.71%
Region: Tohoku	8.00%	8.05%	8.10%	7.12%	7.81%
Region: Kanto	29.63%	30.40%	29.25%	33.40%	30.66%
Region: Chubu	19.72%	18.57%	19.48%	18.10%	18.97%
Region: Kinki	16.48%	17.07%	16.30%	16.65%	16.64%
Region: Chugoku	6.35%	5.81%	6.96%	5.72%	6.19%
Region: Shikoku	3.33%	3.48%	3.72%	2.86%	3.34%
Region: Kyushu	11.80%	11.66%	11.87%	11.38%	11.68%

**Fig. 2:**
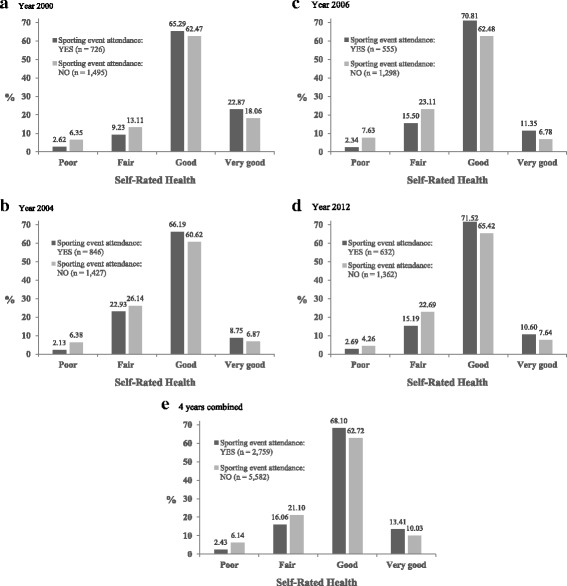
Distribution of Self-Rated Health Categories for Sporting Event Attendees and Non-Sporting Event Attendees

Figure 2 illustrates the data distribution for each category of self-rated health for sporting event attendees (i.e., respondents attending at least one sporting event during the past year) and non-sporting event attendees (i.e., respondents not attending any sporting event during the past year), respectively. As shown in the figure, sporting event attendees were more likely to report good or very good health than non-sporting event attendees for all 4 years. In particular, based on the 4-years combined data, 82% of the sporting event attendees were classified into the categories of good or very good health, compared to 73% of the non-sporting event attendees. These results provided preliminary evidence of the association of sporting event attendance with enhanced self-rated health.

Table 2 provides the results of the two-level multilevel ordinal logistic regression analysis. Region-level variances were small across the 4 years, indicating that the effect of region was minimal. Controlling for the effects of the control variables, sporting event attendance had a significant positive association with self-rated health for all 4 years, with an unstandardized coefficient of 0.28 (*p* = .003; 95% CI = [.09, .47]) for 2000, 0.25 (*p* = .002; 95% CI = [.09, .41]) for 2004, 0.49 (*p* < .001; 95% CI = [.32, .67]) for 2006, and 0.39 (*p* < .001; 95% CI = [.24, .55]) for 2012. Except for sport participation level, sporting event attendance was the only variable that significantly explained self-rated health for all 4 years. In addition, the last column of Table 2 presents regression estimates based on the 4-years combined data. After controlling for the year effects (Year 2000 was set as a reference category), sporting event attendance was positively associated with self-rated health (*B* = 0.33, *p* < .001; 95% CI = [.28, .39]). Importantly, the coefficient estimates for sporting event attendance indicated respondents attending at least one sporting event during the past year were 33% more likely (based on the 4-years combined data) to report a higher level of self-rated health than those who did not attend any sporting event. These results provided support for the hypothesis, suggesting that sporting event attendance has a positive association with self-rated health across the observation period.

**Table 2:** Results of Multilevel Ordinal Logistic Regression Analysis Explaining Self-Rated Health

	2000		2004		2006		2012		4 years combined	
Variable	B	SE	B	SE	B	SE	B	SE	B	SE
Sporting event attendance	0.28^**^	0.10	0.25^**^	0.08	0.49^***^	0.09	0.39^***^	0.08	0.33^***^	0.03
Age	−0.03^***^	0.00	−0.01^*^	0.00	0.00	0.00	−0.02^**^	0.01	−0.01^***^	0.00
Male	0.10	0.11	0.03	0.13	−0.02	0.16	−0.25^***^	0.05	−0.03	0.08
Living with a spouse	0.32^*^	0.13	−0.01	0.07	0.16	0.09	0.08	0.09	0.13^**^	0.05
Unemployment	−0.42^**^	0.13	−0.57^***^	0.10	−0.72^***^	0.19	−0.23	0.24	−0.47^***^	0.10
Sport participation level	0.11^**^	0.04	0.20^***^	0.04	0.23^***^	0.03	0.16^***^	0.02	0.17^***^	0.01
City size	−0.02	0.05	−0.05	0.03	0.02	0.05	0.03	0.05	0.01	0.02
Year effects^a^										
2004									−0.87^***^	0.11
2006									−0.70^***^	0.06
2012									−0.63^***^	0.10
Region-level variance	0.02	0.02	0.01	0.01	0.01	0.01	0.00	0.00	0.01	0.00
*N*	2221		2273		1853		1994		8341	

*B*: unstandardized coefficients. *SE*: robust standard error

^a^Year 2000 was set as a reference category

* p < 0.05, ** p < 0.01,*** p < 0.001

## Discussion

Building upon Grossman’s [9] health production model and psychological research on the health benefits of psychosocial resources [11, 12], the current research examined the association between sporting event attendance and self-rated health using multiyear cross-sectional data from the NSLS, a national survey conducted in Japan. The results offer support for the positive relationship between sporting event attendance and self-rated health over a 12-year period, extending the understanding of how the perception of health relates to nonexercise forms of leisure [6, 7, 25, 28]. This study also contributes to the literature examining the relationship between sport spectatorship and health outcomes [17, 18, 29–31] by revealing that sporting event attendance correlates with self-rated health—a robust predictor of mortality risk.

Our results indicated that sporting event attendance had a large and meaningful association with self-rated health. As discussed above, when compared to individuals who did not attend any sporting event during the past year, those who attended one or more events were 33% more likely to indicate a higher level of self-rated health. An alternative view suggests that the association between sport spectatorship and health outcomes is created by another characteristic that correlates with both variables [30]. The use of a cross-sectional research design does not allow us to disregard this view; still, it is important to note that we confirmed the hypothesized relationship by controlling for some personal (such as age, employment status, and sport participation) and environmental (such as location of residence) characteristics that may establish this relationship. Consequently, our finding, together with evidence from past research [14, 15, 17, 18], lends support to the claim that sporting event attendance could be associated with psychosocial health resources, which in turn contribute to maintaining and enhancing health as measured by an elevated level of self-rated health.

Japan represented an ideal context for testing the hypothesized relationship between sporting event attendance and self-rated health for the following reasons. First, the country has invested substantial resources in spectator sport events and venues. It hosted several mega-sporting events, including the 1964 Tokyo Olympics, the 1972 Sapporo Winter Olympics, the 1998 Nagano Winter Olympics, and the 2002 FIFA World Cup (co-hosted with South Korea), and has been selected as the host country for the 2020 Tokyo Olympics. In addition to these one-time events, the country is host to numerous professional sport leagues and events, including baseball, football, basketball, golf, and sumo wrestling, with amateur events (especially collegiate and high school sports) also enjoying high popularity. Second, during the study period (2000–2012), the country experienced two important social events that could impact the strength of this relationship: the co-hosting of the FIFA World Cup in 2002 and the Great East Japan Earthquake in 2011. Existing evidence indicated that hosting mega sporting events or facing major disasters would temporarily increase the health function of spectator sport [15]. Thus, if the relationship between sporting event attendance and self-rated health is a temporal phenomenon, this relationship would be more evident during the period following those events (2004 and 2012 in the current study) than during other periods (2000 and 2006 herein). However, the examination of the regression coefficients across the years reveals a different pattern, where the largest coefficient for sporting event attendance was observed in 2006. Consequently, the current analysis of multiyear data in Japan would suggest that the strength of the association between sporting event attendance and self-rated health does not depend on the occurrence of significant social events.

The use of a multilevel analysis represents another strength for this study. In the NSLS data, individual responses (at the first level) were nested within each geographic region of Japan where respondents lived (at the second level). For this hierarchical structure, the assumption of independent observations may be violated and lead to the underestimation of standard errors if a conventional ordinary least square regression is used [32]. For example, areas in each region in Japan share similar characteristics in terms of weather, economic and labor conditions, access to healthcare systems, and access to sporting events and venues, and these similarities may systematically influence the responses of individuals from the same region. The multilevel analysis used for this study accounted for these potential dependencies in the datasets and allowed us to provide unbiased estimates regardless of the presence of the dependencies.

Despite the aforementioned strengths, some limitations of this research should be noted. First, because of the cross-sectional nature of the study, causality between sporting event attendance and self-rated health cannot be inferred. Future research should use a longitudinal research design or identify appropriate instrumental variables to gain insight on causal inference.

Second, this research examined the association between sporting event attendance and a subjective evaluation of health. Although self-rated health represents a valid health indicator that can predict mortality risk [8, 10], using objective health data (e.g., body mass index, blood pressure, mortality, biomarkers) should enable future research to strengthen confidence in this study’s findings.

Third, given the use of probability sampling methods for the NSLS (e.g., two-stage stratified random sampling in 2000, 2004, and 2006), the current findings would be generalizable to the adult population in Japan. Still, our findings may be subject to nonresponse bias [33] because a portion of individuals selected as initial study participants did not respond to the survey. This limitation does not allow us to exclude the possibility that subjects who failed to offer responses “are different than those who [responded to the survey] on the characteristics of interest in the study” [34].

Fourth, because of this study’s focus on Japanese adults, we are unable to apply the findings to other countries. It is important to test the hypothesized relationship using data collected from other countries to assess the applicability of our evidence.

Fifth, although we controlled for respondents’ sport participation level in assessing the relationship between sporting event attendance and self-rated health, previous research indicated other types of leisure may also correlate with health outcomes [6, 7]. Future researchers are encouraged to investigate the extent to which sporting event attendance is associated with self-rated health, controlling for various other leisure alternatives. They should further determine how the strength of this association compares with that of the correlations between self-rated health and other leisure options.

Sixth, this study hypothesized a positive relationship between sporting event attendance and self-rated health based on the notion that attending sporting events may contribute to creating various psychosocial resources, such as positive emotions and social support. However, some evidence suggests those resources may also be attained through other behavioral means to follow sport teams and events, such as watching games on television [29, 35]. Consequently, it would be essential to explore how sport spectatorship may differently correlate with self-rated health, depending on specific means of sport consumption (e.g., attending live games, watching games at home on television).

Finally, building on Grossman’s [9] health production model and psychological research on the health benefits of psychosocial resources [11, 12], the introduction section of this article provides a plausible explanation of why the relationship between sporting event attendance and self-rated health exists. However, the proposed mechanism underlying this relationship remains untested and requires empirical scrutiny. Future researchers should conduct a field survey to measure constructs representing psychosocial resources (e.g., positive emotions, meaning, friendship), along with sporting event attendance and self-rated health, and assess the extent to which these constructs mediate the relationship observed in the current study.

## Conclusions

In conclusion, the current multiyear investigation of the relationship between sporting event attendance and self-rated health in Japan provides evidence that attending live sporting events is associated with better self-rated health over time. Our findings offer correlational support for the notion that attending sporting events represents an important nonexercise form of leisure that might produce psychosocial resources and contribute to good health. It is hoped that the current evidence will encourage further research efforts that seek to advance the potential role of spectator sport in creating healthier communities.

## Abbreviation

NSLS: National Sports-Life Survey

## Footnotes

1 The surveys conducted before 2000 were excluded from the current analysis because these surveys measured self-rated health using 3-point (1998) or 5-point scales (1992, 1994, 1996), inconsistent with the 4-point scale used for the surveys conducted in the four years examined for this study (2000, 2004, 2006, and 2012).
